# Sustainability of the Healthy Municipalities Strategy in Guatemala^*^

**DOI:** 10.26633/RPSP.2021.70

**Published:** 2021-06-11

**Authors:** Jorge Laureano-Eugenio, Raúl Otoniel Gómez-Rodríguez, Jhunny Tasejo-Corzantes, Augusto Silvestre Ramírez, Rosa María Pretell Aguilar, Jacqueline Elizabeth Alcalde-Rabanal

**Affiliations:** 1 Ministry of Health Jalisco Mexico Ministry of Health, Jalisco, Mexico; 2 Ministry of Health and Social Welfare Guatemala City Guatemala Ministry of Health and Social Welfare, Guatemala City, Guatemala.; 3 Ministry of Health Lima Peru Ministry of Health, Lima, Peru.; 4 National Institute of Health Public Morelos Mexico National Institute of Health Public, Morelos, Mexico.

**Keywords:** Evaluation study, healthy city, health promotion, local government, Guatemala, Estudio de evaluación, ciudad saludable, promoción de la salud, gobierno local, Guatemala, Estudo de avaliação, cidade saudável, promoção da saúde, governo local, Guatemala

## Abstract

**Objective.:**

Evaluate the sustainability of the Healthy Municipalities strategy in Guatemala in order to have solid evidence to support decision-making.

**Methods.:**

A concurrent mixed-methods study was carried out in five phases: 1) theoretical-conceptual (based on a narrative review of the literature on sustainability, dimensions and categories were proposed for evaluation); 2) empirical (four municipalities were selected for convenience and 29 semi-structured interviews and four focus groups were conducted with key actors to explore sustainability; with this information, a score was assigned to each category and dimension); 3) analytical, by category and dimension (content analysis was performed for qualitative information, and totals and averages were calculated for quantitative information); 4) integrative (qualitative data were integrated into matrices by category and dimension, and quantitative data were supported by qualitative information); and 5) meta-inference (consideration was given to the context and its influence on the results).

**Results.:**

Ninety-two (92) informants participated. In operational terms, progress was observed in the transfer and use of results, and in rotations in leadership. In the legal and political sphere, accountability and local planning were highlighted. In the economic sphere, progressive investment in health, water and sanitation was emphasized, as well as insufficient investment in social determinants of health. In the social sphere, few mechanisms were observed to promote and strengthen social participation.

**Conclusions.:**

In the municipalities that participated in the study, a fair level of sustainability was observed in the Healthy Municipalities strategy.

## RESUMEN

**Objetivo.:**

Evaluar la sostenibilidad de la estrategia Municipios Saludables en Guatemala, para disponer de evidencia sólida que permita apoyar la toma de decisiones.

**Métodos.:**

Se realizó un estudio mixto concurrente en cinco fases: 1) teórica-conceptual, se realizó una revisión narrativa de literatura en sostenibilidad, con base en la cual se propusieron dimensiones y categorías para su evaluación; 2) empírica, se seleccionaron por conveniencia cuatro municipios y se realizaron 29 entrevistas semiestructuradas y cuatro grupos focales a actores clave para explorar la sostenibilidad; con esta información, se asignó un puntaje a cada categoría y dimensión; 3) analítica, por categoría y dimensión, se realizó el análisis del contenido para la información cualitativa y sumatorias y promedios para la información cuantitativa; 4) integrativa, los datos cualitativos se integraron en matrices por categoría y dimensión; y los datos cuantitativos se respaldaron con información cualitativa; y 5) metainferencia, se reflexionó sobre el contexto y su influencia en los resultados.

**Resultados.:**

Participaron 92 informantes. En la dimensión operativa, se destacan avances en la transferencia y el uso de resultados, así como en el relevo de liderazgo. En la dimensión legal y política, se destacaron la rendición de cuentas y los planes locales. En la dimensión económica, se destacó la inversión progresiva en salud, agua y saneamiento, con deficiencia de inversiones en determinantes sociales de la salud. En la dimensión social, se observaron pocos mecanismos para impulsar y fortalecer la participación social.

**Conclusiones.:**

En los municipios que participaron del estudio se observó un nivel regular de sostenibilidad de la estrategia Municipios Saludables.

## RESUMO

**Objetivo.:**

Avaliar a sustentabilidade da estratégia de Municípios Saudáveis na Guatemala para dispor de evidências sólidas para apoiar o processo decisório.

**Métodos.:**

Foi realizado um estudo de método misto concorrente em cinco fases: 1) fase teórica-conceitual em que foi feita a revisão narrativa da literatura em sustentabilidade a partir da qual foram propostas dimensões e categorias a serem avaliadas; 2) fase empírica em que foi feita a seleção por conveniência de quatro municípios, com 29 entrevistas semiestruturadas e quatro grupos de discussão com as principais partes interessadas para explorar a sustentabilidade; a partir das informações coletadas, foi dada uma pontuação a cada categoria e dimensão; 3) fase analítica, por categoria e dimensão, em que foi realizada a análise do conteúdo para os dados qualitativos e feito o cálculo de somatórias e médias para os dados quantitativos; 4) fase integrativa em que os dados qualitativos foram integrados em matrizes por categoria e dimensão e os dados quantitativos foram respaldados com a informação qualitativa e 5) metainferência em que foi analisado o contexto e sua influência nos resultados.

**Resultados.:**

O estudo incluiu 92 participantes. Na dimensão operacional, destacam-se os avanços na transferência e no uso de resultados, bem como a ênfase em liderança. Na dimensão jurídico-política, destacam-se a prestação de contas e os planos locais. Na dimensão econômica, destaca-se o investimento progressivo em saúde, água e saneamento, com investimento inadequado nos determinantes sociais da saúde. Na dimensão social, observam-se poucos mecanismos para incentivar e reforçar a participação social.

**Conclusões.:**

Foi observado nos municípios participantes do estudo um nível constante de sustentabilidade da estratégia de Municípios Saudáveis.

The Healthy Municipalities (*Municipios Saludables*) initiative is promoted by the Pan American Health Organization (PAHO) and the World Health Organization (WHO). This initiative is based on the principles of primary health care presented in Alma-Ata in 1978, and on the Ottawa Charter, drafted during the First International Conference on Health Promotion in 1986, which urged promoting the creation of healthy environments (1). The Healthy Municipalities Strategy (HMS) positions health promotion (HP) as its centerpiece and as a priority of the local health policy agenda. It is based on intersectoral action, community participation, and interculturalism (2), which means that it seeks the participation of local government authorities, communities, and other actors (3).

In a healthy municipality, “authorities, institutions, and citizens work together for the health and well-being of its inhabitants, addressing the health determinants that are the responsibility of the government and civil society” (4). In 1992, at the International Conference on Health Promotion and Equity, Guatemala committed to promoting healthy environments (5). In 1999, the Department of Health Promotion was tasked with implementing this policy; this end, committees were set up in the provinces involving municipalities, health sector representatives, and other local organizations. Community health commissions were also formed to promote social participation and health policies at the local level. As part of this policy’s rollout, an assessment was conducted and a Municipal Health Plan was launched to achieve an impact on social and environmental determinants (6, 7).

These initiatives are examples of the efforts to implement the HMS in Guatemala over more than two decades, involving the Ministry of Public Health and Social Assistance, municipalities, and community organizations. The time frame of its implementation raises the question of the level of sustainability of the HMS. We can begin answering this question by defining *sustainability*. In theory, the term refers to an action that can be sustained; that is, which it is possible to maintain over a long period of time (8). From this standpoint, the sustainability of the HMS depends on stakeholders’ capacity to maintain, on an ongoing basis, the strategy’s activities and results. Processes must be developed that respond to the characteristics of the social, cultural, political, and economic context in which an intervention is carried out. A sustainable intervention seeks the continuous improvement of a situation (9), and the HMS is seeking to constantly improve the population’s health conditions (10).

Implementing an intervention requires financial resources and the forging of ties between government actors and civil society, while taking into account the legal and institutional considerations that facilitate its implementation (11). This is why, for an intervention to yield sustainable results, it is essential to establish processes that can be carried out continuously over time (9). Accordingly, sustainability must be analyzed from a holistic perspective. One of the main variables is an intervention’s permanence over time; another is the scope (12) of implementation (operational, political-legal, economic, and social). The study of the *operational dimension* is focused on analyzing institutional technical capabilities (leadership, human resources training, and management) and operational capabilities (availability of human resources, supplies, and materials) (13). The *political-legal dimension* involves the existence of clear and measurable agreements, formalized in advance, that facilitate or hinder implementation (9). The *economic dimension* considers the availability of economic investment for implementing an intervention in the medium and long term (14). Finally, the *social dimension* analyzes society’s active and critical involvement in interventions, as well as its legitimacy to demand and propose actions for the strategy’s continuity (15).

No previous studies were found in the literature regarding the sustainability of the HMS. Therefore, the present study is important, because it is the first analysis of HMS implementation using a sustainability approach. This paper evaluates the sustainability of the HMS in Guatemala, to provide robust evidence enabling evidence-based decision-making in the Ministry of Health and Social Assistance and other stakeholder entities, to strengthen the sustainability of the strategy’s results.

## MATERIALS AND METHODS

**FIGURE 1.**
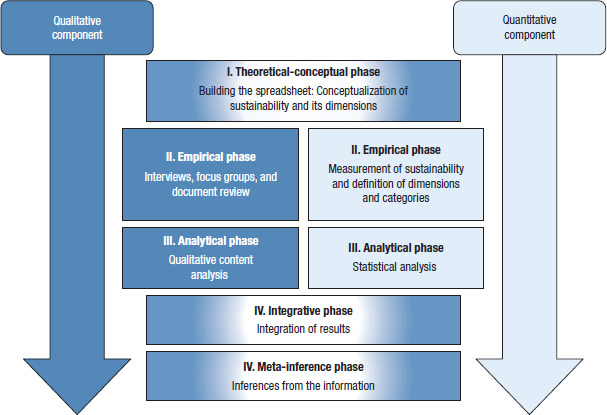
Phases of the study

***Source:*** Prepared by the authors.

An evaluation was conducted on the sustainability of the HMS in Guatemala from 2015 to 2019, using a concurrent mixed (qualitative and quantitative) design (16). The phases of the study are described below (figure 1).

1. **Theoretical-conceptual phase.** Given the lack of previous studies evaluating the interventions’ sustainability, the first task was to identify the dimensions for evaluating it. To this end, a narrative review of the literature on sustainability in HP was conducted. The search included publications from the last five years for Latin America and the Caribbean, carried out using the PubMed, LILACS, and Google Academic search engines. The search terms in English were “sustainability”, “strategy continuity”, and “strategy monitoring”; their Spanish-language counterparts were, respectively, “*sostenibilidad*”, “*continuidad de estrategias*” and “*seguimiento de estrategias*”. These were used with Boolean operators (AND, OR), as well as the phrase “health promotion”, or “*promoción de salud*”, in Spanish. A total of 312 articles were found, and their summaries reviewed; 36 of these articles conceptualized or measured sustainability and then presented an in-depth review; 22 of them developed dimensions and categories to evaluate sustainability.

Four dimensions and 22 categories were identified for the evaluation. This proposal was shared and discussed online with seven experts, trained in and with broad experience in HP, from Chile, Guatemala, Mexico, and Peru (two HP directors, two HP researchers, two advisors in HMS implementation, and a national HP coordinator), who considered four dimensions and 18 categories to evaluate sustainability (table 1).

2. **Empirical phase.** Four municipalities were selected, according to the following criteria:
  - implemented the HMS since 2000;
  - received technical and financial support from WHO and the Ministry of Health;
  - implemented projects with local resources;
  - held leading positions in the HMS between 2015-2019; and
  - rural-urban and indigenous-non-indigenous populations.

Each municipality was assigned a letter (A, B, C, or D) to maintain the anonymity of the information.

Twenty-nine semi-structured interviews were conducted with individuals involved in implementing the HMS at the national and municipal levels. Interviewers used an outline that probed operational, political-legal, financial, and social dimensions of sustainability. Snowball sampling was used, and interviews were halted when new information stopped appearing in the categories. At the national level, coordinators of government institutions in charge of the HMS were interviewed. At the municipal level, interviewees included those implementing the strategy (mayors, town council members, or delegates), representatives of other institutions (coordinators of government were interviewed, civil society organizations, international entities), and representatives of the population (community health commissions, community development committees, and social leaders).

**TABLE 1.** Categories and dimensions for the study on sustainability in health promotion

Dimensions	Categories
1. Operational sustainability	1.1. Availability of staff 1.2. Transfer and use of results 1.3. Intersectoral action 1.4. Rotation in leadership 1.5. Monitoring and follow-up 1.6. Evaluation of the strategy
2. Political-legal sustainability	2.1. Local agreements and legal arrangements 2.2. Local plans using a sustainable development and HP approach 2.3. Guidelines for implementation of the strategy 2.4. Accountability
3. Economic sustainability	3.1. Economic resources for health, water, and sanitation 3.2. Economic resources for operational actions 3.3. Economic resources for evaluation
4. Social sustainability	4.1. Active involvement of the population 4.2. Acceptability of the strategy 4.3. Autonomy and proactive capabilities of the population 4.4. Use of innovation and social interaction technologies 4.5. Level of impact

***Source:*** Prepared by the authors.

Four focus groups (one in each municipality) explored the dimensions of HMS sustainability, with nine to 16 informants (including coordinators of government institutions, civil society organizations, international organizations, community health commissions, community development committees, and social leaders). The interviews and focus groups were conducted according to schedules agreed in advance with the informants, in spaces that ensured their privacy. Before the groups started meeting, participants signed a consent form. The interviews and focus groups were recorded and transcribed with a word processor. Reports on the HMS, plans, and evaluation reports were also reviewed.

For the quantitative qualification of each dimension of sustainability in each municipality, the first step was to score the categories. Each category was assigned a score from 0 to 5 (0 = very bad, 1 = bad, 2 = fair, 3 = good, and 4 = very good), according to the level of progress. With a view to reaching a consensus on the score for each category, the researchers prepared, with the experts’ assistance, an instrument describing the qualifying criteria for each one.

3. **Analytical phase.** During this phase, the qualitative and quantitative scores were assessed:
  a. Qualitative. The interviews and focus groups were transcribed and the content analysis was carried out according to the defined dimensions and categories (table 1). NVivo Version 12 Bonus® software was used for coding.
  b. Quantitative. Based on the participants’ assessment, scores were given for every category in every municipality, as well as the average overall scores for the four municipalities.
4. **Integrative phase.** Data matrices were organized by dimension and category for each municipality, based on information from interviews, focus groups, and documents. With this information, the researchers individually scored each category for each municipality. These scores were reviewed and discussed with the group of experts and, by consensus, they assigned a final score. The score for each dimension was obtained from the average of scores for their categories. This result was compared with the reports on the matrices, and shared with the informants to validate the score assigned to each category and dimension. These results were expressed in graphs, incorporating the qualitative information.
5. **Meta-inference phase.** The researchers jointly considered their quantitative and qualitative findings on HMS sustainability. The political, social, and cultural context was analyzed, as well as the historical, normative, legal, and institutional referents regarding HP in Guatemala over the last five years to seek responses to the research group’s findings and their variability among the municipalities.

This study was approved by the Ethics Committee of the Ministry of Public Health and Social Assistance of Guatemala and by the Ethical Review Committee of the Pan American Health Organization (PAHOERC).

## RESULTS

Of the 92 participants in the interviews and focus groups, 44 (48%) were men and 48 (52%) women, with ages from 19 to 66 years. The majority had secondary-school studies, and their involvement in HMS implementation ranged from one to six years. The majority belonged to civil society organizations (table 2).

### Operational sustainability

**FIGURE 2.**
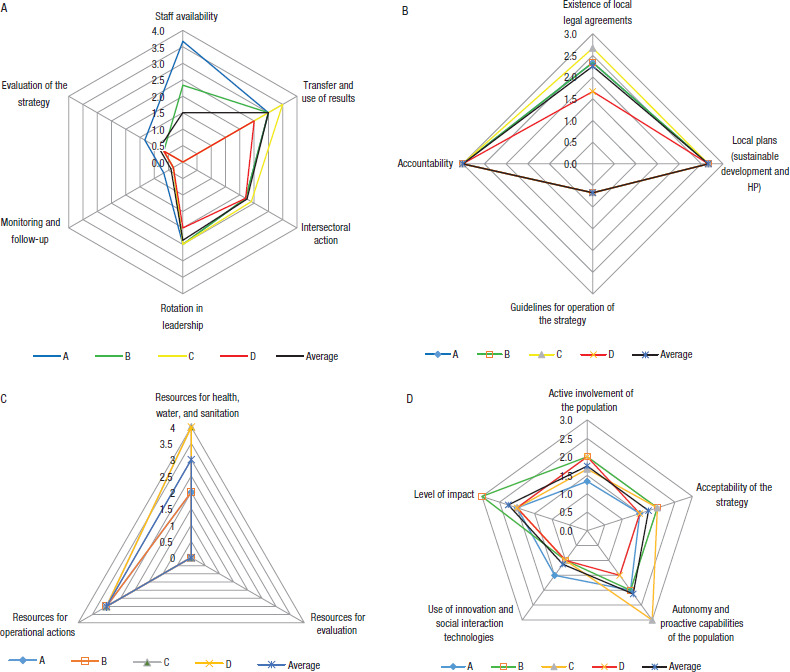
Dimensions of sustainability: a) operational, b) political-legal, c) economic, d) social

In evaluating the operational dimension of sustainability (figure 2a), the progress observed differed from one municipality to another. The greatest progress was identified in the transfer and use of results and the rotation in leadership; these categories were scored 3 and 2.5, respectively. The greatest availability of staff for implementation of the strategy was found in municipality A; availability in municipality D was very bad. Municipalities A and B included HP staff in their organizational structure, whereas municipalities C and D did not, and only took into account municipal representatives or health commissions.

**TABLE 2.** Participants in interviews and focus groups of the Healthy Municipalities Strategy in Guatemala, 2019

Municipality	Mayor	Municipal official	Municipal MSPAS^b^ official	Departmental MSPAS^b^ official	National official	Other agencies and civil society^c^	Total
A	1	5	2	2		20	30
B		3	2	2		11	18
C	1	4	2	2		10	19
D	1	4	2	2		11	20
E^a^					5		5
Total	3	16	8	8	5	52	92

a National level

b Ministry of Public Health and Social Assistance (*Ministerio de Salud Pública y Asistencia Social*)

c Representatives of local development committees, civil society groups, community leaders, international cooperation organizations

In the transfer and use of results, municipality C showed the most progress, and municipality D the least. Regarding intersectoral action and rotation in leadership, all the municipalities showed the same performance level, with scores ranging from 2 to 2.5. Intersectoral action in municipalities A and B is carried out between the Town Council and the Ministry of Health; in municipalities C and D, international cooperation agencies are added.

In three of the four municipalities, it was observed that when the local government changed, HP activities continued. However, in municipality D, the new authorities did not continue the activities launched by the previous administration.

*“…For obviously political reasons, the town has suffered a lot, because it doesn’t belong to the party in power nationally, which makes it hard to continue activities”.* DPM-C

Concerning monitoring, follow-up, and evaluation, all the municipalities have had problems carrying them out. The evaluations that the four municipalities conduct are basically financial, and informants acknowledged that they do not have trained evaluation staff; in these cases, they ask other entities for support.

### Legal and political sustainability

Figure 2b shows that accountability is the category where the most progress has been made, with an average score of 3; existence of local plans scored 2.7; and existence of local agreements, 2.5. The municipality with the most progress in legal and political sustainability is municipality C; the one with the least progress is D. As an accountability exercise, the municipalities publish reports on their quarterly achievements, sharing them with the Municipal Development Council. The local plans and agreements considered projects having an impact on health determinants.

*“…we were given ideas to improve quality of life, such as ordering and organizing housing, hand washing, healthy food for children. Children who grow up malnourished have no possibilities for the future.”* AM-D

Regarding regulation, advances in water and sanitation, land management, and construction stand out, among other areas. No municipality has operational guidelines for the HMS, and the national level does not have documents that serve as a reference to be adapted at the local level.

### Economic sustainability

The national government strengthened procedures for municipal budget planning and execution (figure 2c). Nevertheless, investment regulations were focused on addressing national priorities and did not allocate funds for evaluation. Under these regulations, the four municipalities made steady investments in health, water, and sanitation; however, they faced delays in their execution. Municipalities A and B earmarked part of their municipal budget to training staff for the HMS; municipalities C and D used international cooperation funds. A problem identified for executing the budget was the limited participation of other local actors.

*“…when other government agencies get involved, they don’t finish giving the allocated funds; they give them until the following year, but then they don’t give the full amount.”* EM-B

According to the budget analysis for 2008, 2012, 2016, and 2018, municipality A allocated 79% of its programmed budget (124 703 425 quetzals, equivalent to 16 132 397 United States dollars [US$]) to health, water, and sanitation; municipality B allocated 74% (82 176 895 quetzals, or 10 630 905 US$); municipality D allocated 44% (16 012 917 quetzals, or 2 071 529 US$); and municipality C allocated 135% of its budget (7 845 305 quetzals, or 1 014 917 US$). All of these municipalities executed their budget adequately, pursuant to the regulations, which require executing at least 10% of the budget every year.

### Social sustainability

Municipalities C and D showed the most progress in the social dimension (figure 2d). Regarding mechanisms for active involvement of the population, the participation of the development councils—including municipal authorities, government institutions, and local organizations—has been promoted.

*“…when we want to do a project for the community, we liaise with the COCODES [Community Development Committees], which convene everyone in the neighborhoods where action will be taken; people are very cooperative.”* AM-B

As to the acceptability of the HMS, people are resistant to the implementation of projects that are planned without their input. Projects that include community participation in their design and planning are better accepted, and there is greater demand for their continuity.

With regard to the autonomy and proactive capabilities of the population, in municipalities A and B, HP projects were promoted with the participation of community leaders. In municipality C, HP actions of were promoted by international cooperation agencies and then adopted by the municipal government and development councils. In municipality D, these projects still depend on international cooperation agencies.

*“…NGOs* [nongovernmental organizations] *such as Save the Children… donate materials and resources to carry out a project, and we contribute part of it. Then we explain to people how the project will turn out…”* DPM-A

Regarding the use of technology for innovation and social interaction, the platform of the Assembly of Development Councils is used to disseminate actions implemented at the community level. However, the participation of community leaders in these spaces does not guarantee effective communication to the population and municipal authorities.

**FIGURE 3.**
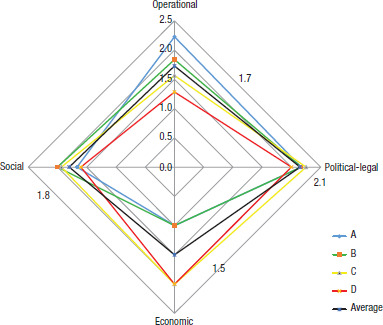
Scores for the four dimensions of sustainability of the Healthy Municipalities Strategy in Guatemala (2019)

The evaluation of the dimensions of sustainability in the four municipalities studied is presented in figure 3; overall, municipality C had the highest level of sustainability. An average score of 2 was found for the operational, social, and economic dimensions; the political-legal dimension scored an average of 2.5. Operational sustainability is negatively affected by deficient monitoring and evaluation processes. The social dimension is impacted by the limited use of innovation and social interaction technologies, and by whether people accept interventions. Regarding the economic dimension, municipalities have improved their budget programming and execution in health, water, and sanitation; however, they face budget problems in financing evaluation. In the political-legal dimension, advances in accountability, local planning, and local agreements were found, although there are regulatory gaps for HMS implementation.

## DISCUSSION

There is an obvious lack of previous studies evaluating the sustainability of interventions. This is the first study to evaluate the sustainability of the HMS in Guatemala, providing empirical and methodological evidence on the dimensions proposed for evaluation.

An important finding regarding the operational dimension of HMS sustainability is the availability of staff in municipality A, which is the municipality with the highest level of operational sustainability. Documented evidence shows that the availability of trained staff improves an intervention’s management and implementation (17). By contrast, municipalities C and D face staff shortages for implementing the HMS. Other countries that implemented this strategy have likewise faced the problem of insufficient human resources with HP competencies. Therefore, having sufficient staff with adequate competencies is a challenge (18), since human resources are essential in implementing any intervention (19, 20). In municipality D, it was observed that the new authorities did not provide continuity to HMS activities; changes in the government and in officials has been documented as a factor limiting operational sustainability (9). The incipient progress found in HMS monitoring and evaluation show the limited priority given to evaluation; this is related, in turn, to deficient management. In contrast to the Guatemalan situation, in Argentina, HMS decisions are based on information from evaluations (21).

The political-legal dimension of sustainability scored the highest, with advances in accountability being especially noteworthy. Less progress was observed in the development of national and municipal guidelines for HMS implementation. A study that analyzed the implementation and sustainability of a health strategy found that having clear and consistent guidelines are a strength for its implementation (22). However, if the regulatory framework does not respond to the context, implementation is a greater challenge, making it necessary to prepare regulations jointly with representatives of civil society and other stakeholders (23).

Economic sustainability was the area that showed the least progress. There were abundant financial resources for water and sanitation; however, the resources for projects in other HP areas were insufficient. This calls into question the approach to social determinants of health, and makes it necessary to convene local actors in order to finance projects that go beyond water and sanitation. The municipalities invested more than 10% in health, meaning they had adequate municipal investment levels. These data contrast with Chilean research showing that investment is lower in the poorest municipalities (24) and that it is insufficient to address health problems. Therefore, in this type of initiatives the participation of international cooperation funds (25) is relevant. With regard to the social dimension of sustainability, major progress was not found. This is partly explained by the absence of effective mechanisms for the participation of stakeholders in HMS design, implementation, evaluation, and monitoring (26). There is a consensus that, in order to act on the social determinants of health, governments must create opportunities to motivate the participation of citizens (27) and social organizations (28). It is likewise recognized that municipal plans having an impact on the social determinants of health are strengthened by social participation (29), favoring the continuity of actions (30). On the contrary, limited social participation limits people’s acceptance of the HMS (22).

Regarding limitations of this study, one is the lack of previous studies to evaluate the sustainability of interventions, which would have made it possible to conduct the evaluations in a shorter period. The informants’ reports on the operational and social dimensions could not be confirmed because the data available in the municipalities is primarily financial.

In summary, the HMS has attained what can be considered a fair level of sustainability in the Guatemalan municipalities studied. Within the operational dimension, improvement is needed in the availability of operational staff, monitoring, and evaluation. In the political-legal dimension, there is a clear need to define guidelines for implementation. In the economic dimension, improvement in local consensus-building is necessary to attract more resources and finance projects having an impact on the social determinants of health. In the social dimension, it is essential to implement strategies that facilitate social and community participation by the general population.

## Declaration.

The sponsors did not participate in any way in the study’s design, the collection and analysis of data, the decision to publish this work, or the preparation of the manuscript. Authors hold sole responsibility for the views expressed in the manuscript, which may not necessarily reflect the opinion or policy of the *RPSP/PAJPH* and/or of PAHO.
